# A second monoclinic polymorph for 3-amino-1-(4-meth­oxy­phen­yl)-9,10-dihydro­phenanthrene-2,4-dicarbonitrile

**DOI:** 10.1107/S1600536812011798

**Published:** 2012-03-24

**Authors:** Abdullah M. Asiri, Hassan M. Faidallah, Khalid A. Alamry, Seik Weng Ng, Edward R. T. Tiekink

**Affiliations:** a Chemistry Department, Faculty of Science, King Abdulaziz University, PO Box 80203, Jeddah, Saudi Arabia; b The Center of Excellence for Advanced Materials Research, King Abdulaziz University, Jeddah, PO Box 80203, Saudi Arabia; c Department of Chemistry, University of Malaya, 50603 Kuala Lumpur, Malaysia

## Abstract

The title compound, C_23_H_17_N_3_O, has been previously described in a monoclinic *P*2_1_/*c* polymorph with *Z* = 4 [Asiri, Al-Youbi, Faidallah, Ng & Tiekink (2011). *Acta Cryst*. E**67**, o2449]. In the new monoclinic *P*2_1_/*n* form, with *Z* = 8, there are two independent mol­ecules, *A* and *B*, in the asymmetric unit. In both mol­ecules, the cyclo­hexa-1,3-diene ring has a screw-boat conformation, whereas it is a distorted half-chair in the original polymorph. There is a fold in each mol­ecule, as indicated by the dihedral angle between the benzene rings of the 1,2-dihydro­naphthalene and aniline residues of 33.19 (10)° (mol­ecule *A*) and 30.6 (10)° (mol­ecule *B*). The meth­oxy­benzene ring is twisted out of the plane of the aniline residue to which it is connected [dihedral angles = 49.22 (10) and 73.27 (10)°, in *A* and *B* respectively]. In the crystal, the two independent mol­ecules self-associate *via* N—H⋯N hydrogen bonds, generating a 12-membered {⋯HNC_3_N}_2_ synthon. These are connected into a supra­molecular tape in the (-101) plane by N—H⋯O(meth­oxy) inter­actions. In the *P*2_1_/*c* polymorph, supra­molecular layers are formed by N—H⋯N and N—H⋯O inter­actions.

## Related literature

For background to the biological activity of related phenanthrene compounds, see: Wang *et al.* (2010 ▶); Rostom *et al.* (2011 ▶). For related structures, see: Asiri *et al.* (2011*a* ▶,*b* ▶); Al-Youbi *et al.* (2012 ▶). For ring puckering parameters, see: Cremer & Pople (1975 ▶).
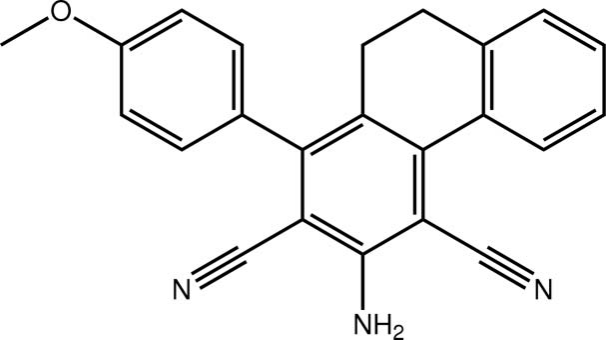


## Experimental

### 

#### Crystal data

C_23_H_17_N_3_O
*M* *_r_* = 351.40
Monoclinic, 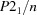

*a* = 11.5197 (6) Å
*b* = 25.1585 (12) Å
*c* = 11.9564 (6) Å
β = 90.719 (5)°
*V* = 3464.9 (3) Å^3^
*Z* = 8
Mo *K*α radiation
μ = 0.09 mm^−1^
*T* = 100 K
0.35 × 0.35 × 0.10 mm

#### Data collection

Agilent SuperNova Dual diffractometer with an Atlas detector
Absorption correction: multi-scan (*CrysAlis PRO*; Agilent, 2011 ▶) *T*_min_ = 0.971, *T*_max_ = 0.992
20450 measured reflections
7990 independent reflections
5348 reflections with *I* > 2σ(*I*)
*R*_int_ = 0.055

#### Refinement

*R*[*F* ^2^ > 2σ(*F* ^2^)] = 0.060
*wR*(*F* ^2^) = 0.156
*S* = 1.04
7990 reflections
503 parameters
4 restraints
H atoms treated by a mixture of independent and constrained refinement
Δρ_max_ = 0.33 e Å^−3^
Δρ_min_ = −0.25 e Å^−3^

### 

Data collection: *CrysAlis PRO* (Agilent, 2011 ▶); cell refinement: *CrysAlis PRO*; data reduction: *CrysAlis PRO*; program(s) used to solve structure: *SHELXS97* (Sheldrick, 2008 ▶); program(s) used to refine structure: *SHELXL97* (Sheldrick, 2008 ▶); molecular graphics: *ORTEP-3* (Farrugia, 1997 ▶) and *DIAMOND* (Brandenburg, 2006 ▶); software used to prepare material for publication: *publCIF* (Westrip, 2010 ▶).

## Supplementary Material

Crystal structure: contains datablock(s) global, I. DOI: 10.1107/S1600536812011798/bt5850sup1.cif

Structure factors: contains datablock(s) I. DOI: 10.1107/S1600536812011798/bt5850Isup2.hkl

Supplementary material file. DOI: 10.1107/S1600536812011798/bt5850Isup3.cml

Additional supplementary materials:  crystallographic information; 3D view; checkCIF report

## Figures and Tables

**Table 1:** Hydrogen-bond geometry (Å, °)

*D*—H⋯*A*	*D*—H	H⋯*A*	*D*⋯*A*	*D*—H⋯*A*
N2—H2⋯N6	0.89 (1)	2.25 (2)	3.081 (3)	157 (3)
N5—H3⋯N1	0.88 (1)	2.36 (1)	3.213 (3)	162 (2)
N2—H1⋯O1^i^	0.88 (2)	2.56 (2)	3.271 (3)	139 (2)

Symmetry code: (i) 
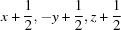
.

**Fig. 1.**
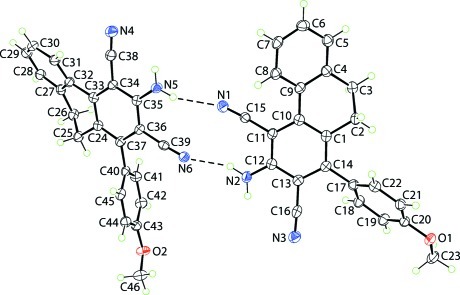
The molecular structure of (I) showing the atom-labelling scheme and displacement ellipsoids at the 50% probability level.

**Fig. 2.**
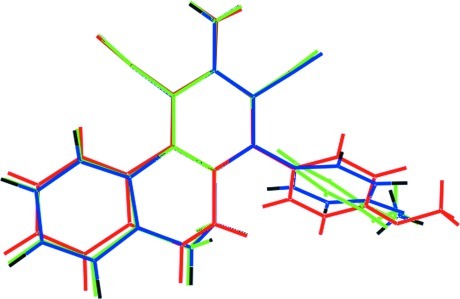
Overlay diagram of the two independent molecules of (I) and the polymorph of (I). The molecules have been aligned so that nitrile-N atom closest to the partially saturated ring, amino-N and the C atom diagonally opposite to the amino-N atom have been superimposed. Colour code: red = N1-molecule of (I), green = N4-molecule (inverted) of (I) and blue = polymorph of (I).

**Fig. 3.**
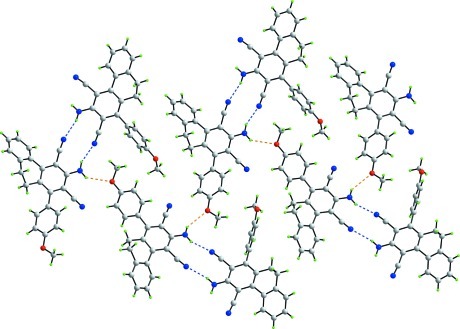
A view of the supramolecular tape along [1 0 1] in (I). The N—H···N and N—H···O hydrogen bonds are shown as blue and orange dashed lines, respectively.

## supplementary crystallographic information

### Comment

In connection with structural studies on phenanthrene compounds (Asiri *et al.*, 2011*a*; Asiri *et al.*, 2011*b*; Al-Youbi *et al.*, 2012), of interest owing to biological activity (Wang *et al.* 2010; Rostom *et al.*, 2011), a new monoclinic polymorph of the title compound 3-amino-1-(4-methoxyphenyl)-9,10-dihydrophenanthrene-2,4-dicarbonitrile (I) was found. Previously (I) was isolated in the monoclinic space group *P*2_1_/*c* with *Z* = 4 (Asiri *et al.*, 2011*b*). The new form crystallizes in the monoclinic space group *P*2_1_/*n* with two independent molecules in the asymmetric unit, Fig. 1.

The conformations of the two independent molecules in (I) differ in two regions of the molecule. In the N1-containing molecule, the cyclohexa-1,3-diene ring has a screw-boat conformation as defined by the following geometric parameters (Cremer & Pople, 1975): puckering parameters q_2_ = 0.527 (2) Å and q_3_ = 0.156 (2) Å, and amplitudes: Q = 0.549 (2) Å, θ = 73.5 (2)° and φ_2_ = 93.2 (3)°. By contrast, in the N4-containing molecule, the conformation is based on a distorted half-chair with puckering parameters: q_2_ = 0.530 (2) Å, q_3_ = -0.178 (2) Å, Q = 0.558 (2) Å, θ = 108.5 (2)° and φ_2_ = 265.8 (3) °. In the *P*2_1_/*c* polymorph of (I), the conformation matches more closely a distorted half-chair.

For the first independent molecule, the benzene rings of the 1,2-dihydronaphthalene and methoxybenzene residues form dihedral angles of 33.19 (10) and 49.22 (10)°, respectively, with the amino-benzene ring, indicating non-planarity in the fused ring system and a twist of the methoxybenzene out of the plane of the benzene ring to which it is connected. The comparable angles for the second independent molecule are 30.6 (10) and 73.27 (10)°, respectively. Figure 2 shows an overlay diagram for the three independent molecules of (I) characterized in the two polymorphs.

In the crystal structure of (I) the two independent molecules self-associate *via* N—H···N hydrogen bonds to generate 12-membered {···HNC_3_N}_2_ synthons, Fig. 3 and Table 1. One of the amino-H atoms forms a hydrogen bond to a methoxy-O atom leading to the formation of a supramolecular tape along [1 0 1], Fig. 3 and Table 1. The fourth independent amino-H atom does not participate in a significant intermolecular interaction. In the previously described *P*2_1_/*c* form of (I), supramolecular arrays with a zigzag topology were formed through N—H···N hydrogen bonds, leading to {···HNC_3_N}_2_ synthons, as well N—H···O(methoxy) hydrogen bonding.

### Experimental

A mixture of 4-methoxybenzaldehyde (1.38 g, 0.01 mmol), 1-tetralone (1.46 g, 0.01 mmol), malononitrile (0.66 g, 0.01 mmol) and ammonium acetate (6.2 g, 0.08 mmol) in absolute ethanol (50 ml) was refluxed for 6 h. The reaction mixture was allowed to cool, and the formed precipitate was filtered, washed with water, dried and recrystallized from ethanol. Yield: 69%. M.pt: 533–535 K.

### Refinement

Carbon-bound H-atoms were placed in calculated positions [C—H = 0.95 to 0.99 Å, *U*_iso_(H) = 1.2 to 1.5*U*_eq_(C)] and were included in the refinement in the riding model approximation. The N—H atoms were located in a difference Fourier map, and were refined with a distance restraint of N—H = 0.88±0.01 Å; their *U*_iso_ values were refined. Owing to poor agreement, the (1 0 1) and (0 2 1) reflections were omitted from the final cycles of refinement.

### Crystal data

**Table:**

C_23_H_17_N_3_O	*F*(000) = 1472
*M**_r_* = 351.40	*D*_x_ = 1.347 Mg m^−^^3^
Monoclinic, *P*2_1_/*n*	Mo *K*α radiation, λ = 0.71073 Å
Hall symbol: -P 2yn	Cell parameters from 4701 reflections
*a* = 11.5197 (6) Å	θ = 2.4–27.5°
*b* = 25.1585 (12) Å	µ = 0.09 mm^−^^1^
*c* = 11.9564 (6) Å	*T* = 100 K
β = 90.719 (5)°	Prism, orange
*V* = 3464.9 (3) Å^3^	0.35 × 0.35 × 0.10 mm
*Z* = 8	

### Data collection

**Table:**

Agilent SuperNova Dual diffractometer with an Atlas detector	7990 independent reflections
Radiation source: SuperNova (Mo) X-ray Source	5348 reflections with *I* > 2σ(*I*)
Mirror monochromator	*R*_int_ = 0.055
Detector resolution: 10.4041 pixels mm^-1^	θ_max_ = 27.6°, θ_min_ = 2.4°
ω scan	*h* = −14→14
Absorption correction: multi-scan (*CrysAlis PRO*; Agilent, 2011)	*k* = −24→32
*T*_min_ = 0.971, *T*_max_ = 0.992	*l* = −15→12
20450 measured reflections	

### Refinement

**Table:**

Refinement on *F*^2^	Primary atom site location: structure-invariant direct methods
Least-squares matrix: full	Secondary atom site location: difference Fourier map
*R*[*F*^2^ > 2σ(*F*^2^)] = 0.060	Hydrogen site location: inferred from neighbouring sites
*wR*(*F*^2^) = 0.156	H atoms treated by a mixture of independent and constrained refinement
*S* = 1.04	*w* = 1/[σ^2^(*F*_o_^2^) + (0.057*P*)^2^ + 0.839*P*] where *P* = (*F*_o_^2^ + 2*F*_c_^2^)/3
7990 reflections	(Δ/σ)_max_ = 0.001
503 parameters	Δρ_max_ = 0.33 e Å^−^^3^
4 restraints	Δρ_min_ = −0.25 e Å^−^^3^

### Fractional atomic coordinates and isotropic or equivalent isotropic displacement parameters (Å^2^)

**Table:**

	*x*	*y*	*z*	*U*_iso_*/*U*_eq_	
O1	0.10070 (14)	0.15244 (6)	0.28185 (14)	0.0303 (4)	
O2	0.84581 (15)	0.39154 (6)	1.09183 (14)	0.0328 (4)	
N1	0.35813 (17)	0.54827 (8)	0.59073 (16)	0.0301 (5)	
N2	0.44427 (18)	0.41933 (8)	0.60868 (17)	0.0286 (4)	
N3	0.45795 (18)	0.28657 (8)	0.52814 (17)	0.0341 (5)	
N4	0.48229 (19)	0.77085 (8)	0.78737 (19)	0.0377 (5)	
N5	0.49102 (17)	0.64209 (8)	0.72209 (16)	0.0268 (4)	
N6	0.56385 (16)	0.51011 (7)	0.73917 (15)	0.0249 (4)	
C1	0.15965 (18)	0.40545 (8)	0.38717 (18)	0.0220 (5)	
C2	0.04533 (18)	0.40182 (9)	0.32242 (19)	0.0242 (5)	
H2A	0.0596	0.4063	0.2415	0.029*	
H2B	0.0102	0.3664	0.3338	0.029*	
C3	−0.03757 (19)	0.44473 (8)	0.36222 (19)	0.0256 (5)	
H3A	−0.1106	0.4432	0.3178	0.031*	
H3B	−0.0566	0.4386	0.4417	0.031*	
C4	0.01701 (19)	0.49843 (9)	0.34957 (18)	0.0235 (5)	
C5	−0.0443 (2)	0.54234 (9)	0.31252 (19)	0.0275 (5)	
H5	−0.1252	0.5393	0.2974	0.033*	
C6	0.0111 (2)	0.59115 (9)	0.29700 (19)	0.0292 (5)	
H6	−0.0323	0.6216	0.2748	0.035*	
C7	0.1298 (2)	0.59477 (9)	0.31422 (18)	0.0268 (5)	
H7	0.1686	0.6273	0.2992	0.032*	
C8	0.1925 (2)	0.55150 (9)	0.35315 (18)	0.0249 (5)	
H8	0.2738	0.5546	0.3653	0.030*	
C9	0.13701 (19)	0.50318 (8)	0.37478 (17)	0.0222 (5)	
C10	0.19815 (18)	0.45619 (8)	0.41975 (18)	0.0220 (5)	
C11	0.29307 (18)	0.46056 (8)	0.49625 (18)	0.0218 (5)	
C12	0.35534 (19)	0.41555 (8)	0.53271 (18)	0.0225 (5)	
C13	0.32176 (19)	0.36595 (8)	0.48802 (18)	0.0225 (5)	
C14	0.22155 (19)	0.36049 (8)	0.41905 (18)	0.0211 (5)	
C15	0.32687 (18)	0.51051 (9)	0.54559 (18)	0.0221 (5)	
C16	0.3947 (2)	0.32124 (9)	0.51117 (18)	0.0256 (5)	
C17	0.18686 (18)	0.30606 (8)	0.38398 (18)	0.0228 (5)	
C18	0.1762 (2)	0.26602 (9)	0.46277 (19)	0.0268 (5)	
H18	0.1886	0.2742	0.5396	0.032*	
C19	0.1478 (2)	0.21416 (9)	0.4326 (2)	0.0278 (5)	
H19	0.1418	0.1872	0.4879	0.033*	
C20	0.12838 (18)	0.20240 (9)	0.3206 (2)	0.0248 (5)	
C21	0.13611 (18)	0.24202 (9)	0.24038 (19)	0.0248 (5)	
H21	0.1213	0.2339	0.1639	0.030*	
C22	0.16527 (18)	0.29310 (9)	0.27145 (18)	0.0242 (5)	
H22	0.1708	0.3199	0.2159	0.029*	
C23	0.1006 (2)	0.11064 (9)	0.3630 (2)	0.0355 (6)	
H23A	0.0808	0.0770	0.3263	0.053*	
H23B	0.0430	0.1185	0.4204	0.053*	
H23C	0.1777	0.1078	0.3980	0.053*	
C24	0.69808 (19)	0.63961 (8)	1.01349 (18)	0.0218 (5)	
C25	0.7659 (2)	0.64074 (9)	1.12243 (18)	0.0259 (5)	
H25A	0.8455	0.6540	1.1092	0.031*	
H25B	0.7717	0.6043	1.1535	0.031*	
C26	0.7052 (2)	0.67681 (9)	1.20495 (18)	0.0262 (5)	
H26A	0.6265	0.6630	1.2203	0.031*	
H26B	0.7496	0.6780	1.2763	0.031*	
C27	0.69672 (18)	0.73163 (9)	1.15568 (18)	0.0233 (5)	
C28	0.71185 (19)	0.77700 (9)	1.22026 (19)	0.0269 (5)	
H28	0.7195	0.7738	1.2992	0.032*	
C29	0.71595 (19)	0.82680 (9)	1.1717 (2)	0.0290 (5)	
H29	0.7253	0.8575	1.2172	0.035*	
C30	0.7064 (2)	0.83184 (9)	1.0572 (2)	0.0279 (5)	
H30	0.7140	0.8658	1.0233	0.033*	
C31	0.68564 (19)	0.78737 (9)	0.99132 (19)	0.0252 (5)	
H31	0.6764	0.7912	0.9127	0.030*	
C32	0.67824 (18)	0.73687 (8)	1.03971 (19)	0.0229 (5)	
C33	0.65311 (18)	0.68813 (8)	0.97334 (18)	0.0219 (5)	
C34	0.58428 (18)	0.68879 (8)	0.87531 (18)	0.0213 (5)	
C35	0.56000 (18)	0.64163 (8)	0.81431 (18)	0.0210 (5)	
C36	0.61077 (18)	0.59420 (8)	0.85423 (18)	0.0213 (5)	
C37	0.67812 (19)	0.59281 (8)	0.95346 (18)	0.0215 (5)	
C38	0.5298 (2)	0.73604 (9)	0.8308 (2)	0.0275 (5)	
C39	0.58701 (18)	0.54667 (9)	0.79306 (18)	0.0215 (5)	
C40	0.72664 (19)	0.54034 (8)	0.99069 (17)	0.0209 (5)	
C41	0.6805 (2)	0.51357 (9)	1.08273 (19)	0.0266 (5)	
H41	0.6193	0.5293	1.1237	0.032*	
C42	0.7230 (2)	0.46448 (9)	1.11451 (19)	0.0282 (5)	
H42	0.6908	0.4467	1.1769	0.034*	
C43	0.81266 (19)	0.44099 (9)	1.05564 (18)	0.0244 (5)	
C44	0.8609 (2)	0.46710 (9)	0.96590 (19)	0.0253 (5)	
H44	0.9237	0.4517	0.9266	0.030*	
C45	0.81622 (19)	0.51622 (8)	0.93386 (18)	0.0240 (5)	
H45	0.8482	0.5337	0.8710	0.029*	
C46	0.9325 (2)	0.36427 (10)	1.0298 (2)	0.0368 (6)	
H46A	0.9475	0.3295	1.0640	0.055*	
H46B	1.0041	0.3852	1.0303	0.055*	
H46C	0.9052	0.3593	0.9525	0.055*	
H1	0.4837 (17)	0.3901 (6)	0.6221 (18)	0.020 (6)*	
H2	0.466 (2)	0.4511 (6)	0.633 (2)	0.044 (8)*	
H3	0.469 (2)	0.6121 (6)	0.690 (2)	0.037 (7)*	
H4	0.460 (2)	0.6715 (6)	0.695 (2)	0.034 (7)*	

### Atomic displacement parameters (Å^2^)

**Table:**

	*U*^11^	*U*^22^	*U*^33^	*U*^12^	*U*^13^	*U*^23^
O1	0.0352 (9)	0.0210 (8)	0.0348 (9)	−0.0020 (7)	−0.0035 (8)	−0.0052 (7)
O2	0.0406 (10)	0.0253 (9)	0.0326 (9)	0.0105 (7)	0.0036 (8)	0.0064 (7)
N1	0.0324 (11)	0.0285 (11)	0.0292 (11)	0.0001 (9)	−0.0087 (9)	−0.0013 (9)
N2	0.0327 (11)	0.0217 (11)	0.0312 (11)	−0.0024 (9)	−0.0098 (9)	−0.0009 (9)
N3	0.0395 (12)	0.0264 (11)	0.0360 (12)	0.0011 (10)	−0.0081 (10)	0.0000 (9)
N4	0.0426 (12)	0.0272 (11)	0.0431 (13)	0.0031 (10)	−0.0126 (11)	0.0005 (10)
N5	0.0344 (11)	0.0204 (11)	0.0254 (10)	0.0015 (9)	−0.0075 (9)	0.0019 (9)
N6	0.0275 (10)	0.0229 (10)	0.0240 (10)	0.0010 (8)	−0.0055 (8)	0.0010 (8)
C1	0.0230 (11)	0.0243 (11)	0.0187 (10)	−0.0033 (9)	0.0022 (9)	−0.0005 (9)
C2	0.0256 (11)	0.0244 (11)	0.0226 (11)	−0.0036 (9)	−0.0024 (10)	−0.0009 (9)
C3	0.0243 (11)	0.0280 (12)	0.0246 (11)	−0.0025 (9)	−0.0003 (10)	−0.0016 (10)
C4	0.0249 (11)	0.0267 (12)	0.0190 (11)	0.0006 (9)	0.0006 (9)	−0.0039 (9)
C5	0.0280 (12)	0.0300 (13)	0.0245 (12)	−0.0007 (10)	−0.0009 (10)	−0.0031 (10)
C6	0.0370 (13)	0.0264 (12)	0.0243 (12)	0.0070 (10)	−0.0035 (11)	−0.0011 (10)
C7	0.0352 (13)	0.0240 (12)	0.0213 (11)	−0.0032 (10)	−0.0014 (10)	−0.0053 (9)
C8	0.0301 (12)	0.0242 (12)	0.0204 (11)	−0.0010 (9)	−0.0024 (10)	−0.0037 (9)
C9	0.0270 (11)	0.0222 (11)	0.0174 (10)	0.0000 (9)	0.0013 (9)	−0.0037 (9)
C10	0.0220 (11)	0.0244 (11)	0.0199 (11)	−0.0014 (9)	0.0044 (9)	−0.0006 (9)
C11	0.0222 (11)	0.0205 (11)	0.0228 (11)	−0.0030 (9)	0.0025 (9)	−0.0018 (9)
C12	0.0233 (11)	0.0238 (11)	0.0205 (11)	−0.0027 (9)	0.0033 (9)	0.0002 (9)
C13	0.0269 (11)	0.0213 (11)	0.0194 (11)	−0.0003 (9)	0.0017 (9)	0.0018 (9)
C14	0.0255 (11)	0.0200 (11)	0.0180 (10)	−0.0019 (9)	0.0035 (9)	−0.0004 (9)
C15	0.0218 (11)	0.0222 (11)	0.0221 (11)	0.0009 (9)	−0.0009 (9)	−0.0005 (9)
C16	0.0327 (12)	0.0219 (12)	0.0220 (11)	−0.0069 (10)	−0.0011 (10)	−0.0020 (9)
C17	0.0223 (11)	0.0224 (11)	0.0238 (11)	−0.0005 (9)	0.0010 (10)	−0.0008 (9)
C18	0.0326 (12)	0.0253 (12)	0.0225 (11)	−0.0028 (10)	−0.0016 (10)	−0.0028 (10)
C19	0.0330 (12)	0.0224 (12)	0.0279 (12)	−0.0025 (10)	0.0010 (11)	0.0024 (10)
C20	0.0212 (11)	0.0221 (11)	0.0311 (12)	−0.0010 (9)	0.0008 (10)	−0.0064 (10)
C21	0.0227 (11)	0.0276 (12)	0.0239 (11)	−0.0003 (9)	−0.0008 (10)	−0.0044 (10)
C22	0.0241 (11)	0.0273 (12)	0.0212 (11)	0.0005 (9)	0.0033 (10)	0.0007 (9)
C23	0.0446 (15)	0.0202 (12)	0.0415 (15)	−0.0017 (11)	−0.0057 (13)	−0.0033 (11)
C24	0.0239 (11)	0.0224 (11)	0.0191 (11)	−0.0022 (9)	0.0000 (9)	0.0012 (9)
C25	0.0321 (12)	0.0221 (11)	0.0232 (12)	−0.0008 (9)	−0.0065 (10)	−0.0004 (9)
C26	0.0326 (12)	0.0269 (12)	0.0190 (11)	−0.0045 (10)	−0.0032 (10)	0.0012 (9)
C27	0.0222 (11)	0.0253 (12)	0.0223 (11)	−0.0014 (9)	0.0005 (10)	−0.0021 (9)
C28	0.0280 (12)	0.0277 (12)	0.0250 (12)	0.0024 (10)	−0.0025 (10)	−0.0041 (10)
C29	0.0259 (12)	0.0286 (12)	0.0323 (13)	0.0041 (10)	−0.0031 (11)	−0.0106 (10)
C30	0.0296 (12)	0.0191 (11)	0.0349 (13)	0.0007 (9)	−0.0021 (11)	−0.0006 (10)
C31	0.0272 (11)	0.0239 (12)	0.0246 (12)	0.0012 (9)	−0.0014 (10)	−0.0003 (10)
C32	0.0210 (11)	0.0214 (11)	0.0266 (12)	−0.0008 (9)	0.0033 (10)	−0.0022 (9)
C33	0.0227 (11)	0.0238 (11)	0.0195 (11)	−0.0012 (9)	0.0031 (9)	0.0002 (9)
C34	0.0242 (11)	0.0191 (11)	0.0206 (11)	−0.0007 (9)	0.0009 (9)	0.0009 (9)
C35	0.0222 (11)	0.0222 (11)	0.0187 (11)	−0.0015 (9)	0.0016 (9)	−0.0004 (9)
C36	0.0250 (11)	0.0187 (11)	0.0201 (11)	−0.0016 (9)	0.0016 (9)	−0.0011 (9)
C37	0.0263 (11)	0.0199 (11)	0.0183 (10)	−0.0016 (9)	0.0019 (9)	0.0028 (9)
C38	0.0295 (12)	0.0256 (12)	0.0274 (12)	0.0002 (10)	−0.0029 (10)	−0.0034 (10)
C39	0.0212 (11)	0.0242 (11)	0.0193 (10)	0.0030 (9)	0.0003 (9)	0.0037 (9)
C40	0.0256 (11)	0.0199 (11)	0.0170 (10)	−0.0032 (9)	−0.0042 (9)	−0.0005 (9)
C41	0.0278 (12)	0.0273 (12)	0.0248 (11)	0.0025 (10)	0.0029 (10)	0.0012 (10)
C42	0.0332 (13)	0.0277 (12)	0.0240 (12)	−0.0001 (10)	0.0045 (11)	0.0057 (10)
C43	0.0285 (12)	0.0224 (11)	0.0223 (11)	0.0008 (9)	−0.0043 (10)	0.0018 (9)
C44	0.0263 (11)	0.0261 (12)	0.0236 (11)	0.0002 (9)	0.0022 (10)	−0.0038 (10)
C45	0.0301 (12)	0.0226 (11)	0.0191 (11)	−0.0052 (9)	0.0007 (10)	0.0010 (9)
C46	0.0420 (14)	0.0291 (13)	0.0392 (15)	0.0112 (11)	0.0017 (13)	0.0003 (11)

### Geometric parameters (Å, º)

**Table:**

O1—C20	1.376 (3)	C20—C21	1.387 (3)
O1—C23	1.431 (3)	C21—C22	1.378 (3)
O2—C43	1.370 (3)	C21—H21	0.9500
O2—C46	1.427 (3)	C22—H22	0.9500
N1—C15	1.148 (3)	C23—H23A	0.9800
N2—C12	1.364 (3)	C23—H23B	0.9800
N2—H1	0.879 (9)	C23—H23C	0.9800
N2—H2	0.887 (10)	C24—C37	1.396 (3)
N3—C16	1.153 (3)	C24—C33	1.408 (3)
N4—C38	1.153 (3)	C24—C25	1.511 (3)
N5—C35	1.351 (3)	C25—C26	1.518 (3)
N5—H3	0.884 (10)	C25—H25A	0.9900
N5—H4	0.881 (10)	C25—H25B	0.9900
N6—C39	1.152 (3)	C26—C27	1.503 (3)
C1—C14	1.388 (3)	C26—H26A	0.9900
C1—C10	1.405 (3)	C26—H26B	0.9900
C1—C2	1.522 (3)	C27—C28	1.388 (3)
C2—C3	1.521 (3)	C27—C32	1.406 (3)
C2—H2A	0.9900	C28—C29	1.382 (3)
C2—H2B	0.9900	C28—H28	0.9500
C3—C4	1.499 (3)	C29—C30	1.378 (3)
C3—H3A	0.9900	C29—H29	0.9500
C3—H3B	0.9900	C30—C31	1.387 (3)
C4—C5	1.381 (3)	C30—H30	0.9500
C4—C9	1.416 (3)	C31—C32	1.399 (3)
C5—C6	1.398 (3)	C31—H31	0.9500
C5—H5	0.9500	C32—C33	1.487 (3)
C6—C7	1.383 (3)	C33—C34	1.407 (3)
C6—H6	0.9500	C34—C35	1.419 (3)
C7—C8	1.384 (3)	C34—C38	1.443 (3)
C7—H7	0.9500	C35—C36	1.410 (3)
C8—C9	1.399 (3)	C36—C37	1.410 (3)
C8—H8	0.9500	C36—C39	1.426 (3)
C9—C10	1.474 (3)	C37—C40	1.499 (3)
C10—C11	1.421 (3)	C40—C45	1.383 (3)
C11—C12	1.407 (3)	C40—C41	1.401 (3)
C11—C15	1.440 (3)	C41—C42	1.380 (3)
C12—C13	1.410 (3)	C41—H41	0.9500
C13—C14	1.417 (3)	C42—C43	1.389 (3)
C13—C16	1.429 (3)	C42—H42	0.9500
C14—C17	1.486 (3)	C43—C44	1.381 (3)
C17—C18	1.386 (3)	C44—C45	1.391 (3)
C17—C22	1.404 (3)	C44—H44	0.9500
C18—C19	1.391 (3)	C45—H45	0.9500
C18—H18	0.9500	C46—H46A	0.9800
C19—C20	1.387 (3)	C46—H46B	0.9800
C19—H19	0.9500	C46—H46C	0.9800
			
C20—O1—C23	116.44 (18)	H23A—C23—H23B	109.5
C43—O2—C46	117.81 (17)	O1—C23—H23C	109.5
C12—N2—H1	116.5 (15)	H23A—C23—H23C	109.5
C12—N2—H2	119.4 (18)	H23B—C23—H23C	109.5
H1—N2—H2	123 (2)	C37—C24—C33	119.9 (2)
C35—N5—H3	121.0 (18)	C37—C24—C25	122.62 (19)
C35—N5—H4	122.4 (17)	C33—C24—C25	117.50 (19)
H3—N5—H4	116 (2)	C24—C25—C26	109.51 (19)
C14—C1—C10	120.3 (2)	C24—C25—H25A	109.8
C14—C1—C2	121.95 (19)	C26—C25—H25A	109.8
C10—C1—C2	117.62 (19)	C24—C25—H25B	109.8
C3—C2—C1	109.93 (18)	C26—C25—H25B	109.8
C3—C2—H2A	109.7	H25A—C25—H25B	108.2
C1—C2—H2A	109.7	C27—C26—C25	108.80 (17)
C3—C2—H2B	109.7	C27—C26—H26A	109.9
C1—C2—H2B	109.7	C25—C26—H26A	109.9
H2A—C2—H2B	108.2	C27—C26—H26B	109.9
C4—C3—C2	110.02 (18)	C25—C26—H26B	109.9
C4—C3—H3A	109.7	H26A—C26—H26B	108.3
C2—C3—H3A	109.7	C28—C27—C32	119.2 (2)
C4—C3—H3B	109.7	C28—C27—C26	122.0 (2)
C2—C3—H3B	109.7	C32—C27—C26	118.74 (19)
H3A—C3—H3B	108.2	C29—C28—C27	121.1 (2)
C5—C4—C9	119.7 (2)	C29—C28—H28	119.5
C5—C4—C3	122.7 (2)	C27—C28—H28	119.5
C9—C4—C3	117.7 (2)	C30—C29—C28	119.9 (2)
C4—C5—C6	120.8 (2)	C30—C29—H29	120.1
C4—C5—H5	119.6	C28—C29—H29	120.1
C6—C5—H5	119.6	C29—C30—C31	120.1 (2)
C7—C6—C5	119.4 (2)	C29—C30—H30	120.0
C7—C6—H6	120.3	C31—C30—H30	120.0
C5—C6—H6	120.3	C30—C31—C32	120.6 (2)
C6—C7—C8	120.6 (2)	C30—C31—H31	119.7
C6—C7—H7	119.7	C32—C31—H31	119.7
C8—C7—H7	119.7	C31—C32—C27	118.9 (2)
C7—C8—C9	120.5 (2)	C31—C32—C33	122.7 (2)
C7—C8—H8	119.7	C27—C32—C33	118.35 (19)
C9—C8—H8	119.7	C24—C33—C34	119.70 (19)
C8—C9—C4	118.7 (2)	C24—C33—C32	117.69 (19)
C8—C9—C10	123.2 (2)	C34—C33—C32	122.59 (19)
C4—C9—C10	118.07 (19)	C33—C34—C35	121.59 (19)
C1—C10—C11	119.1 (2)	C33—C34—C38	123.69 (19)
C1—C10—C9	118.7 (2)	C35—C34—C38	114.69 (19)
C11—C10—C9	122.21 (19)	N5—C35—C36	121.38 (19)
C12—C11—C10	121.5 (2)	N5—C35—C34	121.51 (19)
C12—C11—C15	116.2 (2)	C36—C35—C34	117.11 (19)
C10—C11—C15	122.23 (19)	C35—C36—C37	121.83 (19)
N2—C12—C13	121.0 (2)	C35—C36—C39	117.39 (19)
N2—C12—C11	121.7 (2)	C37—C36—C39	120.71 (19)
C13—C12—C11	117.3 (2)	C24—C37—C36	119.81 (19)
C12—C13—C14	121.6 (2)	C24—C37—C40	122.10 (19)
C12—C13—C16	117.7 (2)	C36—C37—C40	118.09 (18)
C14—C13—C16	120.6 (2)	N4—C38—C34	173.6 (2)
C1—C14—C13	119.5 (2)	N6—C39—C36	175.8 (2)
C1—C14—C17	122.6 (2)	C45—C40—C41	117.9 (2)
C13—C14—C17	117.90 (19)	C45—C40—C37	121.21 (18)
N1—C15—C11	175.0 (2)	C41—C40—C37	120.86 (19)
N3—C16—C13	176.7 (2)	C42—C41—C40	120.6 (2)
C18—C17—C22	117.8 (2)	C42—C41—H41	119.7
C18—C17—C14	120.3 (2)	C40—C41—H41	119.7
C22—C17—C14	121.90 (19)	C41—C42—C43	120.3 (2)
C17—C18—C19	121.9 (2)	C41—C42—H42	119.8
C17—C18—H18	119.1	C43—C42—H42	119.8
C19—C18—H18	119.1	O2—C43—C44	124.35 (19)
C20—C19—C18	119.0 (2)	O2—C43—C42	115.62 (19)
C20—C19—H19	120.5	C44—C43—C42	120.0 (2)
C18—C19—H19	120.5	C43—C44—C45	119.1 (2)
O1—C20—C19	123.6 (2)	C43—C44—H44	120.5
O1—C20—C21	116.1 (2)	C45—C44—H44	120.5
C19—C20—C21	120.2 (2)	C40—C45—C44	122.0 (2)
C22—C21—C20	120.1 (2)	C40—C45—H45	119.0
C22—C21—H21	119.9	C44—C45—H45	119.0
C20—C21—H21	119.9	O2—C46—H46A	109.5
C21—C22—C17	121.0 (2)	O2—C46—H46B	109.5
C21—C22—H22	119.5	H46A—C46—H46B	109.5
C17—C22—H22	119.5	O2—C46—H46C	109.5
O1—C23—H23A	109.5	H46A—C46—H46C	109.5
O1—C23—H23B	109.5	H46B—C46—H46C	109.5
			
C14—C1—C2—C3	142.7 (2)	C37—C24—C25—C26	−137.3 (2)
C10—C1—C2—C3	−33.8 (3)	C33—C24—C25—C26	42.0 (3)
C1—C2—C3—C4	57.0 (2)	C24—C25—C26—C27	−59.1 (2)
C2—C3—C4—C5	139.7 (2)	C25—C26—C27—C28	−141.3 (2)
C2—C3—C4—C9	−38.9 (3)	C25—C26—C27—C32	35.7 (3)
C9—C4—C5—C6	1.5 (3)	C32—C27—C28—C29	−3.9 (3)
C3—C4—C5—C6	−177.0 (2)	C26—C27—C28—C29	173.1 (2)
C4—C5—C6—C7	2.9 (3)	C27—C28—C29—C30	−0.8 (3)
C5—C6—C7—C8	−4.0 (3)	C28—C29—C30—C31	3.9 (3)
C6—C7—C8—C9	0.6 (3)	C29—C30—C31—C32	−2.2 (3)
C7—C8—C9—C4	3.9 (3)	C30—C31—C32—C27	−2.5 (3)
C7—C8—C9—C10	−177.96 (19)	C30—C31—C32—C33	178.4 (2)
C5—C4—C9—C8	−4.9 (3)	C28—C27—C32—C31	5.5 (3)
C3—C4—C9—C8	173.76 (19)	C26—C27—C32—C31	−171.6 (2)
C5—C4—C9—C10	176.82 (19)	C28—C27—C32—C33	−175.32 (19)
C3—C4—C9—C10	−4.5 (3)	C26—C27—C32—C33	7.6 (3)
C14—C1—C10—C11	−7.3 (3)	C37—C24—C33—C34	2.6 (3)
C2—C1—C10—C11	169.22 (19)	C25—C24—C33—C34	−176.74 (18)
C14—C1—C10—C9	173.81 (19)	C37—C24—C33—C32	−179.21 (18)
C2—C1—C10—C9	−9.7 (3)	C25—C24—C33—C32	1.5 (3)
C8—C9—C10—C1	−147.5 (2)	C31—C32—C33—C24	151.2 (2)
C4—C9—C10—C1	30.7 (3)	C27—C32—C33—C24	−27.9 (3)
C8—C9—C10—C11	33.7 (3)	C31—C32—C33—C34	−30.6 (3)
C4—C9—C10—C11	−148.1 (2)	C27—C32—C33—C34	150.2 (2)
C1—C10—C11—C12	5.4 (3)	C24—C33—C34—C35	−0.9 (3)
C9—C10—C11—C12	−175.76 (19)	C32—C33—C34—C35	−179.01 (19)
C1—C10—C11—C15	−171.03 (18)	C24—C33—C34—C38	176.9 (2)
C9—C10—C11—C15	7.8 (3)	C32—C33—C34—C38	−1.2 (3)
C10—C11—C12—N2	−178.0 (2)	C33—C34—C35—N5	177.97 (19)
C15—C11—C12—N2	−1.4 (3)	C38—C34—C35—N5	0.0 (3)
C10—C11—C12—C13	1.6 (3)	C33—C34—C35—C36	−1.9 (3)
C15—C11—C12—C13	178.25 (18)	C38—C34—C35—C36	−179.84 (19)
N2—C12—C13—C14	172.7 (2)	N5—C35—C36—C37	−176.77 (19)
C11—C12—C13—C14	−6.9 (3)	C34—C35—C36—C37	3.1 (3)
N2—C12—C13—C16	−9.5 (3)	N5—C35—C36—C39	0.3 (3)
C11—C12—C13—C16	170.85 (19)	C34—C35—C36—C39	−179.80 (18)
C10—C1—C14—C13	2.2 (3)	C33—C24—C37—C36	−1.4 (3)
C2—C1—C14—C13	−174.18 (19)	C25—C24—C37—C36	177.87 (19)
C10—C1—C14—C17	−178.03 (18)	C33—C24—C37—C40	178.31 (18)
C2—C1—C14—C17	5.6 (3)	C25—C24—C37—C40	−2.4 (3)
C12—C13—C14—C1	5.1 (3)	C35—C36—C37—C24	−1.5 (3)
C16—C13—C14—C1	−172.57 (19)	C39—C36—C37—C24	−178.50 (19)
C12—C13—C14—C17	−174.65 (18)	C35—C36—C37—C40	178.76 (18)
C16—C13—C14—C17	7.6 (3)	C39—C36—C37—C40	1.7 (3)
C1—C14—C17—C18	−129.0 (2)	C24—C37—C40—C45	−108.3 (2)
C13—C14—C17—C18	50.8 (3)	C36—C37—C40—C45	71.4 (3)
C1—C14—C17—C22	51.8 (3)	C24—C37—C40—C41	73.3 (3)
C13—C14—C17—C22	−128.4 (2)	C36—C37—C40—C41	−107.0 (2)
C22—C17—C18—C19	1.5 (3)	C45—C40—C41—C42	−0.5 (3)
C14—C17—C18—C19	−177.7 (2)	C37—C40—C41—C42	177.9 (2)
C17—C18—C19—C20	−0.7 (3)	C40—C41—C42—C43	0.2 (4)
C23—O1—C20—C19	−4.4 (3)	C46—O2—C43—C44	−2.8 (3)
C23—O1—C20—C21	175.8 (2)	C46—O2—C43—C42	176.1 (2)
C18—C19—C20—O1	179.52 (19)	C41—C42—C43—O2	−177.9 (2)
C18—C19—C20—C21	−0.7 (3)	C41—C42—C43—C44	1.0 (4)
O1—C20—C21—C22	−178.95 (18)	O2—C43—C44—C45	177.0 (2)
C19—C20—C21—C22	1.2 (3)	C42—C43—C44—C45	−1.8 (3)
C20—C21—C22—C17	−0.4 (3)	C41—C40—C45—C44	−0.3 (3)
C18—C17—C22—C21	−1.0 (3)	C37—C40—C45—C44	−178.8 (2)
C14—C17—C22—C21	178.30 (19)	C43—C44—C45—C40	1.5 (3)

### Hydrogen-bond geometry (Å, º)

**Table:**

*D*—H···*A*	*D*—H	H···*A*	*D*···*A*	*D*—H···*A*
N2—H2···N6	0.89 (1)	2.25 (2)	3.081 (3)	157 (3)
N5—H3···N1	0.88 (1)	2.36 (1)	3.213 (3)	162 (2)
N2—H1···O1^i^	0.88 (2)	2.56 (2)	3.271 (3)	139 (2)

Symmetry code: (i) *x*+1/2, −*y*+1/2, *z*+1/2.
